# Effects of coaches’ autonomy support on athletes’ aggressive behavior and athlete burnout: verification of the mediating effects of coach-athlete relationship and team efficacy

**DOI:** 10.3389/fpsyg.2024.1388185

**Published:** 2024-07-30

**Authors:** Suk-Kyu Kim, Hunhyuk Choi

**Affiliations:** ^1^Dongguk University WISE, Gyeongju, Republic of Korea; ^2^Kangwon National University, Chuncheon, Republic of Korea

**Keywords:** autonomy support, coach-athlete relationship, team efficacy, aggressive behavior, athlete burnout, team sports, cross-sectional study

## Abstract

**Purpose:**

This study investigated the relationships between perceived autonomy support, coach–athlete relationship, team efficacy, aggressive behavior, and athlete burnout among team sports athletes. It verified the mediating effects of the coach–athlete relationship and team efficacy on the relationship between autonomy support and athlete burnout.

**Design, methodology, and approach:**

A questionnaire survey on autonomy support, coach–athlete relationships, aggressive behavior, and athlete burnout was administered to 336 team sports athletes (292 male athletes and 44 female athletes). A cross-sectional research design was used to collect the data. To analyze the collected data, frequency, reliability, descriptive statistical, and correlation analyses were performed using SPSS version 26.0. In addition, confirmatory factor analysis, convergent validity tests, and structural model analysis were conducted using AMOS version 24.0. Bootstrapping was used to examine the mediating effects.

**Results:**

The fit of the measurement model was assessed by calculating the fit indices as follows: *x*^2^ = 329.689, df = 124, *p* < 0.001, TLI = 0.945, CFI = 0.956, RMSEA = 0.070 (90% CI = 0.061–0.080), and SRMR = 0.060. Autonomy support positively affected the coach–athlete relationship (*β* = 0.841) and team efficacy (*β* = 0.338). The coach–athlete relationship positively affected team efficacy (*β* = 0.479). Furthermore, autonomy support did not significantly influence aggressive behavior (*β* = −0.053), and negatively affected athlete burnout (*β* = −0.305). The coach–athlete relationship also did not significantly affect aggressive behavior (*β* = 0.054), and negatively affected athlete burnout (*β* = −0.303). Team efficacy negatively affected aggressive behavior (*β* = −0.516) and athlete burnout (*β* = −0.201). Finally, autonomy support was found to affect athlete burnout through the coach–athlete relationship and team efficacy.

**Conclusion:**

Considering that autonomy support affects athlete burnout through coach–athlete relationship and team efficacy, coaches need to enhance the quality of the coach–athlete relationship and improve team efficacy to reduce athlete burnout. Above all, the study findings suggest that coaches need to provide autonomy-supportive behaviors.

## Introduction

In team sports, if coaches and athletes trust, understand, and communicate with each other and work in unison even in difficult situations, it is ideal for both, individual players’ personal growth and the team’s success (Jung and Choi, 2022).

Coaches must identify, understand, and focus on individual athletes’ strengths (Park et al., 2023). Above all, coaches should acknowledge that athletes’ strengths ultimately add to their team’s competitiveness (Kim and Park, 2020). They should bring out the resources or talent that individual athletes possess, and help athletes display and use their strengths more effectively (Peterson and Seligman, 2004; Lyubomirsky and Layous, 2013; Meyers et al., 2015). From this perspective, coaches’ autonomy support refers to a coaching method that provides athletes with autonomy and leads them to act independently on their own initiative, continuously develop their capabilities by promoting self-directed endeavors, and provides them with choice (Reeve et al., 2004; Bartholomew et al., 2009; Balaguer et al., 2018; Raabe et al., 2019). For example, in training situations, if athletes perceive their coach to have a controlling attitude and provide them with inappropriate feedback (i.e., provision of unclear information), they will have a negative attitude toward the coach, their anxiety level will increase, and motivation level will decrease (Weiss et al., 2009). Autonomy-supportive coaching behavior has been shown to have a negative association with conflicts among members (Weinstein et al., 2016), aggressive behavior (Kim and Choi, 2022), and burnout (Choi et al., 2020; Jowett et al., 2021), and a positive correlation with maintenance of a high-quality coach–athlete relationship, effort, self-esteem, and performance (Bartholomew et al., 2010). Therefore, when coaches choose to engage in autonomy-supportive behavior rather than using control methods to bring out athletes’ best performance and lead the team to victory, they can expect positive outcomes from their team (Kim and Choi, 2022).

In team sports, effective implementation of strategies and tactics is an important factor in determining victory or defeat (Lee and Kim, 2021). Effective communication between the coach and athletes and that among athletes is essential for the flawless execution of tactics (Kim et al., 2020). Furthermore, the formation and maintenance of a close relationship between the coach and athletes serves as the foundation for the success and development of the team (Choi et al., 2018). Nevertheless, in situations where winning games is the top priority of each team, every team inevitably experiences conflicts between athletes and coaches who want to achieve good performance (records). However, it is necessary to overcome these conflicts and work harmoniously. The coach–athlete relationship presented by Jowett and Poczwardowski (2007) is a psychological concept characterized by its interpersonal nature, and was developed as a function of socialization and interaction occurring in a group, such as team efficacy (collective efficacy) and team cohesion (Jowett and Cockerill, 2003). Previous studies on the coach–athlete relationship have demonstrated that this relationship is closely related to sport anger and aggressive behavior (Choi and Cho, 2019), athlete burnout (Isoard-Gautheur et al., 2016; Jung and Choi, 2022), team cohesion and coach leadership (Jowett and Chaundy, 2004), conflicts and support (Jowett, 2009), collective efficacy (Jackson et al., 2009; Hampson and Jowett, 2014), and coach autonomy support in team and individual sports (Raabe et al., 2019; Mossman et al., 2022). According to Jowett et al. (2012), the maintenance of a good coach–athlete relationship is associated with a higher level of team efficacy, and such a relationship has a stronger effect on team efficacy than on team cohesion (e.g., athletes with a high level of satisfaction in team sports were reported to be more likely to contribute to the improvement of team cohesion).

In team sports, collaboration between team members is promoted by team efficacy (Chase et al., 2003). According to Bandura (2000), collective efficacy (team efficacy) is related to the collaborative use of resources possessed by individual members and is a key factor in determining a team’s resilience to adversity, effort level, goal level, and expected performance. From this perspective, Zaccaro et al. (1995) reported that team efficacy has the greatest influence on athletic performance. Additionally, Hampson and Jowett (2014) reported that the manner in which athletes collaborate to achieve a common goal is a key element of team efficacy (Kawazu et al., 2012). In particular, Ramzaninezhad et al. (2009) found that team sports, which are characterized by a high level of interdependence among members, are more closely related to team performance than are individual sports, characterized by a low level of interaction among members. Team members’ strong trust in their team is considered an important factor in successful team outcomes, and good team performance can be expected when team members are interdependent. However, aggressive behavior is one of the major factors that can negatively impact a team’s performance and expectations of winning games. Athletes’ aggressive behavior does not help their team to win a game, and may threaten their athletic career.

In competitive sports, athletes’ negative emotional experiences and aggression are commonly observed phenomena. This aggressive behavior has become a serious problem, attracting the attention of researchers (Sacks et al., 2003). In sports, aggression refers to a behavior performed with the intention of inflicting physical or psychological harm on a person (Silva, 1983). Maxwell and Moores (2007) defined aggression in sports as intentional behavior that causes serious physical or psychological harm to an opponent, regardless of whether it is socially acceptable. Brown and Treviňo (2006) claimed that aggression is not only an individual athlete’s problem but also the team’s problem. From this perspective, Kim and Choi (2022) argued that athletes’ high belief in team efficacy can reduce the frequency of aggressive behaviors and strengthen team members’ collaborative behavior. Therefore, aggressive behavior in sports can be prevented if teammates in a sports team acknowledge each other, behave considerately toward each other, and try to reduce their frustration to reduce or control aggressive behavior in sports (Eagly and Steffen, 1986).

Athletes’ strong trust in their team is considered an important factor in team success, and such beliefs positively influence their ability and confidence. This contributes to a reduction in athlete burnout. Athlete burnout does not simply imply that athletes drop out of their sport, but also refers to a state of psychological and emotional exhaustion (Smith, 1986). According to Raedeke (1997), athlete burnout due to stress comprises three dimensions: a reduced sense of accomplishment, physical and emotional exhaustion, and sport devaluation. The concept of athlete burnout has contributed considerably to our understanding of sports, sports injuries, and sports society (Schmidt and Stein, 1991).

Recent studies on athlete burnout in competitive sports settings have pointed out the importance of cognitive and social factors (Davis et al., 2019). It has been reported that athletes experience burnout as a result of long-term exposure to stress (Wiederhold et al., 2018), which is the most consistent claim in overall studies on burnout (Gustafsson et al., 2017). Nicholls et al. (2007) reported on the differences in the level of stress between individual and team sports athletes, mentioning that athlete burnout is associated with decreased motivation and a decline in performance, and is ultimately related to many negative outcomes, including sports dropout. In particular, an integrated model of athlete burnout presented by Gustafsson et al. (2011) includes antecedents, such as personality, coping, and the environment, early signs, and consequences. Above all, it provides a comprehensive conceptual framework for understanding athlete burnout and analyzing and preventing athletes’ maladaptive psychological outcomes. Westfall et al. (2018) investigated the association between the coach–athlete relationship and the level of burnout among coaches and found that commitment and complementarity, which are subfactors of the coach–athlete relationship, may significantly affect and lower levels of burnout in coaches. Appleby et al. (2018) demonstrated that athletes’ perceptions of burnout and the team’s collective burnout were related to athletes’ individual burnout. More specifically, they claimed that a collective mood may develop among teammates as a result of shared experiences, and teammates may develop similar emotions that may affect athletes’ perceptions. Park and Huh (2018) also showed that a higher level of team efficacy is associated with a lower level of athlete burnout, thus empirically demonstrating that team efficacy plays an important role in preventing athlete burnout.

Taken together, athletes’ aggressive behavior and burnout promote negative emotions, cause athletes to lose interest in and motivation for sports, and reduce expectations of their team’s success. In view of the findings described above, this study sought to investigate the effectiveness of coaches’ autonomy support in reducing athletes’ aggressive behavior and preventing athlete burnout, and examine the roles of the coach–athlete relationship and team efficacy in the relationship between autonomy support, aggressive behavior, and athlete burnout. Therefore, if this study empirically verifies the importance of coaches’ autonomy support, coach–athlete relationship, and team efficacy in reducing athletes’ aggressive behavior and preventing athlete burnout, it will present empirically substantiated data for sports teams’ success that would be useful for coaches and athletes in the field of team sports. Furthermore, Understanding and managing athlete burnout requires a multidisciplinary approach, incorporating fields such as physiology (Beehr and Glaser, 2005), biochemistry (Baum and Posluszny, 1999; Suls and Bunde, 2005), neurology (Schaufeli and Enzmann, 1998; Shirom, 2003), and psychology. Especially, psychological factors such as motivation and stress management play a critical role in the experience of fatigue and athletic performance. Such multifaceted research and approaches provide essential foundations for optimizing athlete training and performance, ultimately helping athletes achieve their peak potential. Continuous research and practical applications are necessary to deepen our understanding of athlete burnout and manage it effectively. Based on these prior findings, we propose the following hypotheses:

*Hypothesis 1*: Autonomy-support coaching will have a positive effect on coach-athlete relationship.

*Hypothesis 2*: Autonomy-support coaching will have a positive effect on team efficacy.

*Hypothesis 3*: Coach-athlete relationship will have a positive effect on on team efficacy.

*Hypothesis 4*: Autonomy-support coaching will have a negative effect on aggressive behavior.

*Hypothesis 5*: Autonomy-support coaching will have a negative effect on athlete burnout.

*Hypothesis 6*: Coach-athlete relationship will have a negative effect on aggressive behavior.

*Hypothesis 7*: Coach-athlete relationship will have a negative effect on athlete burnout.

*Hypothesis 8*: Team efficacy will have a negative effect on aggressive behavior.

*Hypothesis 9*: Team efficacy will have a negative effect on athlete burnout.

*Hypothesis 10*: Autonomy-supportive coaching will have an indirect effect on athlete burnout mediated by coach–athlete relationship and team efficacy.

## Methods

### Participants

This study selected college athletes from college sports teams as the study population. In total, 336 college athletes enrolled in universities in Seoul, Gyeonggi-do, Incheon, Chungcheongnam-do, and Chungcheongbuk-do South Korea were selected as participants for this study using convenience sampling (Jöreskog and Sörbom, 1996). Of them, male athletes accounted for 86.9% (292 participants) and female athletes accounted for 13.1% (44 participants). The mean age of the participants was 21.63 years (*SD* = 1.43). The participants were athletes from seven types of team sports; they included 83 basketball players (24.7%), 14 volleyball players (4.2%), 21 water polo players (6.2%), 86 baseball players (25.6%), 74 soccer players (22.0%), 19 field hockey players (5.7%), and 39 handball players (11.6%). The mean length of the athletic career was 9.45 years (*SD* = 2.36), and 85 athletes (25.3%) had experience playing national sports teams (Table 1).

**Table 1:** Participants’ demographic characteristics.

Variable		*N*	%
Gender	Male	292	86.9
	Female	44	13.1
Type of Sports	Basketball	83	24.7
	Volleyball	14	4.2
	Water polo	21	6.2
	Baseball	86	25.6
	Soccer	74	22.0
	Field Hockey	19	5.7
	Handball	39	11.6
National team sports experience	Yes	85	25.3
	No	251	74.7
Exercise experience	Mean (years) = 9.45 (*SD* = 2.36)		
Age	Mean (age) = 21.63 (*SD* = 1.43)		

### Measure

#### Autonomy support

Autonomy-supportive coaching was assessed using the Korean version of a questionnaire developed by Amorose and Anderson-Butcher (2007). The Korean version has been validated by Kim and Park (2009). This scale was used to assess the athletes’ perceptions of autonomy support provided by the coach. This assessment tool contains six items on autonomy support, with each item rated on a 7-point Likert scale ranging from 1 (strongly disagree) to 7 (strongly agree). Regarding the reliability of the tool, Cronbach’s *α* was calculated as *α* = 0.951.

#### Coach-athlete relationship

The coach–athlete relationship was assessed using the Korean version of the Coach–Athlete Relationship Questionnaire (CART-Q) developed by Jowett and Ntoumanis (2004). The Korean version used in this study (KrCART-Q) was validated for use with Korean athletes and coaches by Kim and Park (2008). This scale was used to assess the athletes’ perceptions of the coach-athlete relationship. This assessment tool for the coach–athlete relationship comprises 11 items on three sub-factors: closeness (four items), commitment (three items), and complementarity (four items). Each item is rated on a 7-point Likert scale ranging from 1 (strongly disagree) to 7 (strongly agree). Cronbach’s α values were *α* = 0.955 for closeness, *α* = 0.912 for commitment, and *α* = 0.944 for complementarity.

#### Team efficacy

The team efficacy of soccer players was assessed using a team efficacy scale developed by Yoo and Lim (2009). This scale was designed to assess the athletes’ perceptions of team efficacy. This scale contains 15 items on four subfactors: team ability (four items), trust in a leader (four items), preparation (three items), and unity (four items). Each item is rated on a 7-point Likert scale ranging from 1 (strongly disagree) to 7 (strongly agree). Cronbach’s *α* values were *α* = 0.818 for ability, *α* = 0.880 for preparation, *α* = 0.941 for trust in a leader, and *α* = 0.927 for unity.

#### Aggressive behavior

Aggressive behavior was measured using a Korean-translated short version of the Competitive Aggressiveness and Anger Scale (CAAS) developed by Maxwell and Moores (2007). Hwang and Park (2012) validated the Korean version of CAAS. This scale was used to assess the athletes’ perceptions of behaviors causing shame, pain, or injury. This scale contains 12 items, including 6 items on aggressiveness and 6 items on anger. Each item is rated on a 5-point Likert scale ranging from 1 (strongly disagree) to 5 (strongly agree). The values of Cronbach’s *α* were *α* = 0.828 for anger and *α* = 0.814 for aggressiveness.

#### Athlete burnout

Athletes’ burnout was measured using the Korean version of the scale developed by Raedeke and Smith (2001). This scale was used to assess the athletes’ psychological symptoms of stress associated with physical or psychological burdens, pre-game anxiety, the results of sports games, and negative interpersonal relationships. Choi et al. (2017) validated the Korean version of this tool. This assessment tool includes 15 items, with 5 items each in three subfactors: reduced accomplishment, physical and emotional exhaustion, and sports devaluation. Regarding the reliability of the scale, Cronbach’s *α* values were *α* = 0.720 for reduced sense of accomplishments, *α* = 0.889 for physical and emotional exhaustion, and α = 0.900 for sport devaluation.

### Procedures

This study was conducted after participation in research ethics education (IRB-KWNU-2023-0-104) implemented by the IRB of the affiliated institution (Kangwon National University). To conduct the questionnaire survey, the researcher personally contacted the coaches of each university and sent the questionnaire via email. After the coaches approved the survey and checked the content of the questionnaire, the researcher personally visited the relevant sports teams with assistant researchers (graduate students) to conduct the survey. Participants voluntarily participated in the questionnaire survey, and the survey was only administered to persons who gave written informed consent after they were given sufficient explanations about the purpose and expected effects of the study and strict maintenance of confidentiality. The survey was conducted among athletes who provided informed consent to participate in the study, and it took approximately 10–15 min for each participant to complete the questionnaire survey. The participants were asked to provide honest and sincere responses to each question during the survey and were given small gifts (e.g., provision of a Starbucks coffee coupon) as a token of appreciation.

### Data analysis

Measurement and structural models were constructed, and the study hypotheses were verified using these models (Bae, 2017). Statistical hypothesis testing was conducted using SPSS 26.0 and Amos 26.0 software. Frequency, reliability, correlation (i.e., 0.10 and 0.20 were considered small, between 0.30 and 0.40 were medium, 0.50 and 0.60 large, and 0.70 and 0.80 were very large; Cohen, 1992), descriptive statistical, and confirmatory factor analyses were conducted, along with convergent validity measurement model verification and structural model analysis to test the hypotheses (Lekwa et al., 2018). Additionally, the mediating effects of the variables were examined using the bootstrapping technique. The level of statistical significance was set at *p* < 0.05.

### Verification of the convergent validity of instruments

Coaches’ autonomy support was set as the independent variable, the coach–athlete relationship and team efficacy were set as mediating variables, and aggressive behavior and athlete burnout were set as dependent variables (Corcoran, 1986; Visser et al., 2005). Before conducting structural model analysis, verification of the measurement model was performed, and verification of the structural model was subsequently conducted. To evaluate the measurement model, convergent validity tests were performed for variables such as autonomy support of coaches, coach–athlete relationship, team efficacy, aggressive behavior, and athlete burnout (Table 2). Specifically, the values of construct reliability (C.R.) for each latent variable ranged from 0.702 to 0.906, surpassing the minimum acceptable level (≥0.70), and the values of average variance extracted (AVE) ranged from 0.503 to 0.753, exceeding the threshold (≥0.50), thus, there was no problem with convergent validity (Fornell and Larcker, 1981).

**Table 2:** Factor loading, SRW, C.R, AVE, and Cronbach’s alpha values of measurement model.

Latent variable	Observed variable	SRW	C.R.	AVE	Cronbach’s alpha
Autonomy-support coaching (ASC)	ASC1	0.835	0.906	0.617	0.951
	ASC2	0.888			
	ASC3	0.901			
	ASC4	0.882			
	ASC5	0.862			
	ASC6	0.844			
Coach–athlete relationship (CAR)	Closeness	0.915	0.901	0.753	0.939
	Commitment	0.884			
	Complementarity	0.942			
Team efficacy	Ability	0.544	0.797	0.503	0.834
	Preparation	0.709			
	Trust in a Leader	0.885			
	Unity	0.819			
Aggressive behavior	Anger	0.639	0.702	0.541	0.839
	Aggressiveness	0.696			
Athlete burnout	Reduced sense of Accomplishment	0.698	0.872	0.696	0.780
	Emotional and Physical Exhaustion	0.683			
	Sport Devaluation	0.830			

SRW, standardized regression weights; C.R, construct reliability; AVE, average variance extracted.

## Results

### Descriptive statistics and correlations between variables

Descriptive statistical analysis of the final data selected through the evaluation of the measurement model was conducted (Table 3). With regard to mean scores, autonomy support (*M* = 5.08) had the highest mean score, followed by closeness (*M* = 5.34), commitment (*M* = 5.02), complementarity (*M* = 3.62), preparation (*M* = 5.50), trust in a leader (*M* = 5.31), unity (*M* = 5.18), ability (*M* = 4.58), anger (*M* = 2.51), aggressiveness (*M* = 2.09), physical and emotional exhaustion (*M* = 2.79), sport devaluation (*M* = 2.29), and reduced accomplishment (*M* = 1.33) in descending order. The values of skewness and kurtosis were in the acceptable range (skewness: ≥0.20, kurtosis: ≥0.30), indicating that the normality assumption of data was satisfied (Gravetter and Wallnau, 2016). In addition, correlation analysis was performed to assess the multicollinearity. The correlation analysis results showed that autonomy support was positively correlated with the coach–athlete relationship (*r* = 0.741) and team efficacy (*r* = 0.641), but was negatively correlated with aggressive behavior (*r* = −0.392) and athlete burnout (*r* = −0.638). In addition, coach–athlete relationship was positively correlated with team efficacy (*r* = 0.763), but negatively correlated with aggressive behavior (*r* = −0.386) and athlete burnout (*r* = −0.651). Team efficacy was negatively correlated with aggressive behavior (*r* = −0.497) and athlete burnout (*r* = −0.590). Therefore, in this study, the correlation coefficients between variables were lower than the threshold of 0.85, as recommended by Kline (2011). Therefore, it was determined that the correlation coefficients were within the acceptable range for multicollinearity (Table 3).

**Table 3:** Descriptive statistics and correlation analysis.

	M	SD	SK	KU	A-S	CAR	T-E	AggB
1. Autonomy-Support	5.08	1.28	−0.381	−0.016				
2. Coach-Athlete Relationship	5.20	1.21	−0.569	0.653	0.741 (0.549)			
3. Team Efficacy	5.14	0.97	−0.351	0.272	0.641 (0.410)	0.763 (0.582)		
4. Aggressive Behavior	2.39	0.66	−0.031	−0.385	−0.392 (0.153)	−0.386 (0.148)	−0.497 (0.247)	
5. Athlete Burnout	2.14	0.48	0.006	−0.381	−0.638 (0.407)	−0.651 (0.423)	−0.590 (0.348)	0.587 (0.344)

### Evaluation of the measurement model

The maximum likelihood method is used to estimate the measurement model. For acceptable threshold levels of the fit indices, the thresholds for the chi-square (*X*^2^), comparative fit index (CFI), Tucker–Lewis index (TLI), root mean square error of approximation (RMSEA), and standardized root mean square residual (SRMR) recommended by Hair et al. (2006) were adopted. Specifically, for TLI and CFI, values (between 0 and 1) greater than 0.90 are generally considered to indicate a good fit, and for RMSEA and SRMR, values (between 0.05 and 0.10) lower than 0.08 indicate an acceptable fit (Bae, 2017). Thus, in this study, autonomy support was set as a latent variable that explains the measurement model. However, in the case of the coach–athlete relationship, team efficacy, aggressive behavior, and athlete burnout, item parceling was conducted based on the subfactors presented in previous studies (Kline, 2011). To assess the fit of the measurement model, the fit indices were calculated as follows: *X*^2^ = 329.689, df = 124, *p* < 0.001, TLI = 0.945, CFI = 0.956, RMSEA = 0.070 (90% CI = 0.061–0.080) and SRMR = 0.060. These fit indices satisfied the threshold criteria.

Table 3 presents the size and direction of each correlation coefficient between the variables and the squared correlation coefficients. Regarding discriminant validity, the AVE value was greater than the inter-construct correlation coefficient squared (*ϕ*^2^), indicating that no problem was found with discriminant validity (AVE>*ϕ*^2^). Therefore, the validity of the research model that this study intended to measure was secured and the measurement model was determined to be appropriate for this study. Thus, the structural model was verified in the next step.

### Relationships between research variables

**Figure 1:**
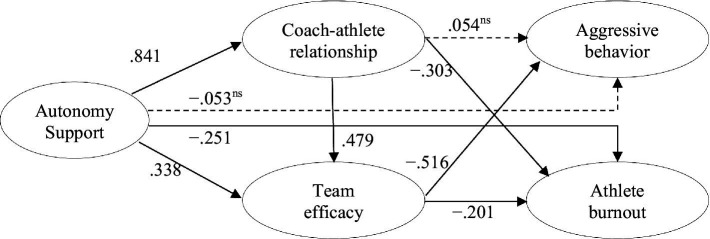
Structural equation modeling of the relationships between autonomy support coaching and athlete burnout. The dotted lines = non-significant paths.

To statistically determine whether to accept or reject the hypotheses in this study, the structural model was verified using the maximum likelihood method (see Figure 1 and Table 4). The analysis results of the fit indices of the structural model were as follows: *X*^2^ = 351.186, df = 125, TLI = 0.940, CFI = 0.951, RMSEA = 0.073 (90% CI = 0.064–0.083), and SRMR = 0.060. Specifically, for TLI and CFI, values greater than 0.90 are generally considered to indicate a good fit, and for RMSEA and SRMR, values lower than 0.08 are considered to indicate an acceptable fit (Bae, 2017). The fit indices satisfied the threshold criteria. Thus, the relationships between the paths between the variables were analyzed, and the results are presented in Figure 1 and Table 4. First, autonomy support exhibited a significant positive effect on the coach–athlete relationship (*β* = 0.841, *p* < 0.001). Second, autonomy support exhibited a significant positive impact on team efficacy (*β* = 0.338, *p* < 0.001). Third, the coach–athlete.

**Table 4:** Path coefficients between latent variables.

Variable		Variable	Estimate	*S.E.*	SRW	C.R.
Autonomy-support	→	Coach–athlete relationship	0.892	0.051	0.841***	17.346
Autonomy-support	→	Team efficacy	0.198	0.054	0.338***	3.655
Coach–athlete relationship	→	Team efficacy	0.264	0.054	0.479***	4.939
Autonomy-support	→	Aggressive behavior	−0.027	0.073	−0.053	−0.371
Autonomy-support	→	Athlete burnout	−0.153	0.071	−0.251*	−2.165
Coach–athlete relationship	→	Aggressive behavior	0.026	0.073	0.054	0.352
Coach–athlete relationship	→	Athlete burnout	−0.174	0.070	−0.303*	−2.471
Team efficacy	→	Aggressive behavior	−0.448	0.120	−0.516***	−3.723
Team efficacy	→	Athlete burnout	−0.209	0.105	−0.201*	−1.990

SRW, standardized regression weights. *p < 0.05, ***p < 0.001.

relationship exhibited a significant positive effect on team efficacy (*β* = 0.479, *p* < 0.01). Fourth, autonomy support did not significantly affect aggressive behavior (*β* = −0.053, *p* > 0.05). Fifth, autonomy support exhibited a significant negative effect on athlete burnout (*β* = −0.251, *p* < 0.05). Sixth, the coach–athlete relationship did not significantly impact aggressive behavior (*β* = 0.054, *p* > 0.05). Seventh, the coach–athlete relationship exhibited a significant negative effect on athlete burnout (*β* = −0.303, *p* < 0.05). Eighth, team efficacy had a significant negative impact on aggressive behavior (*β* = −0.516, *p* < 0.001). Finally, team efficacy had a significant negative effect on athlete burnout (*β* = −0.201, *p* < 0.05).

### Verification of the significance of indirect effects in the structural model

The analysis results of the relationships between the variables can be summarized as follows: First, autonomy support had a positive direct effect on the coach–athlete relationship and team efficacy, but a negative direct effect on athlete burnout. Additionally, the coach–athlete relationship had a positive direct effect on team efficacy, but a negative direct effect on athlete burnout. Furthermore, team efficacy was found to have a direct negative effect on aggressive behaviors and athlete burnout. By verifying the statistical significance of each path between variables, autonomy support was confirmed to have an indirect effect on athlete burnout through the effects of the coach–athlete relationship and team efficacy. These results imply the need for statistical verification of the mediating effects of the coach–athlete relationship and team efficacy in the relationship between autonomy support and athlete burnout (Kline, 2011) (Table 5).

**Table 5:** Direct and indirect effects.

Path	Direct effects	Indirect effects	Total effects
ASC → CAR	0.892	−	0.892***
ASC → T-E	0.198	0.236	0.433***
CAR → T-E	0.264	−	0.264***
CAR → A-B	0.026	−0.118	−0.093
CAR → ABQ	−0.174	−0.055	−0.299*
T-E → A-B	−0.448	−	−0.448***
T-E → ABQ	−0.209	−	−0.209*
^†^ASC → A-B	−0.027	−0.171**	−0.198
^‡^ASC → ABQ	−0.153	−0.246**	−0.399*

ASC, autonomy-support coaching; CAR, coach–athlete relationship; T-E, team efficacy; A-B, aggressive behavior; ABQ, athlete burnout. *p < 0.05, ***p < 0.001.

Therefore, the bootstrapping method (Shrout and Bolger, 2002) was used to verify the statistical significance of the indirect mediating effects of the coach–athlete relationship and team efficacy. Bootstrap resampling was repeatedly performed 2,000 times, and statistical significance was determined using 95% bias-corrected confidence intervals (Table 6). As a result of testing significance of indirect effects through bootstrapping for the paths of autonomy support → coach–athlete relationship → team efficacy → athlete burnout, lower and upper bounds of the 95% bias-corrected confidence intervals were estimated to be −0.436 and −0.089, respectively, and did not contain zero. Indirect effects were estimated to be −0.246 and were statistically significant (*p* < 0.01).

**Table 6:** Results of mediation effects.

Indirect effects	Bootstrap standard errors	Sig	Bootstrapping	
			BC 95% CI Lower bounds	Upper bounds
^†^ −0.246	0.087	0.002**	−0.436	−0.089

**p < 0.01. Bootstrap Sample = 2,000. ^†^Autonomy-support → coach–athlete relationship → team efficacy → athlete burnout.

## Discussion

This study aimed to identify the antecedents of aggression in sports and athlete burnout to reduce aggressive behavior in team sports athletes, develop intervention strategies to prevent or reduce athlete burnout, and provide empirical data in the field of sports. To this end, variables such as autonomy support, coach–athlete relationship, team efficacy, aggressive behavior, and athlete burnout were analyzed, and the results of the analysis are discussed below.

An examination of the relationship between perceived autonomy support and coach–athlete relationships among athletes revealed that autonomy support had a positive effect on the coach–athlete relationship. Coaches’ autonomy-supportive behavior has been reported to positively influence athletes’ emotions. Black and Deci (2000) reported that students’ perceptions of instructors’ autonomy support were associated with higher levels of autonomous self-regulation, perceived competence, interest, and enjoyment, and a lower level of anxiety during the semester. In this respect, coaches will be able to form a more positive relationship and an emotionally strong bond with athletes if they provide the latter with autonomy-supportive coaching. Athletes receiving autonomy support are expected to show improvement in motivation, learning, and performance and experience psychological well-being. Importantly, they are likely to produce outstanding creative and technical outcomes. Some based on self-determination theory (Choi and Huh, 2014; Marcone, 2017; O’Neil and Hodge, 2020) have demonstrated that autonomy-supportive behavior is significantly effective in improving interpersonal relationships in sports situations. Therefore, it is necessary to conduct empirical research on autonomy support in relation to various aspects such as communication, coach–athlete relationships, creativity, and engagement.

This study’s investigation of the relationship between perceived autonomy support and team efficacy among athletes revealed that autonomy support positively affected team efficacy. These results confirm that autonomy-supportive coaching behaviors play an important role in improving team efficacy. Thus, when coaches encourage athletes to think highly of themselves and display belief in their abilities, athletes will work harder to achieve team goals. In connection to this, Høigaard et al. (2015) found that perceived democratic coaching behavior is an important predictor for team efficacy among female handball players. Democratic coaching behavior refers to coaches’ leadership behavior that allows team members to participate in decision-making regarding team goals, training methods, game tactics, and strategies. Therefore, coaches are able to improve perceived team efficacy considerably if they set team goals, systematically establish strategies, tactics, and training processes to achieve team goals, and provide athletes with the same.

In this study, the perceived coach–athlete relationship was found to influence athlete burnout. Thus, a good coach–athlete relationship is important to reduce or prevent athlete burnout. The development of empathy among team members is important to form or maintain a good coach–athlete relationship. In addition, to maintain emotional stability, athletes must form high-quality relationships with their coaches. Shepherd et al. (2006) reported that the absence of a coach–athlete relationship may be linked to interpersonal conflict. For example, in competitive games or training situations, if athletes are exposed to unexpected complex environments and fail to achieve desired results, they are likely to exhibit burnout symptoms. Therefore, athletes should reduce their stress levels by seeking advice to solve problems through continuous conversations with the coach.

With respect to coaching behavior, coaches who teach and train athletes based on effective communication can understand their thoughts, intentions, and emotions; thus, they can provide athletes with emotional stability and a sense of unity as a team, thereby leading athletes’ behaviors and emotions in a positive direction. If athletes maintain a high-quality relationship with their coaches, it may positively impact aspects related to motivation, such as the process of learning or acquiring sports skills. A good coach–athlete relationship may also help athletes cope with negative emotions such as stress, conflict, and burnout. Therefore, athletes should attempt to form and maintain high-quality relationships with their coaches.

The results of this study also revealed that the perceived coach–athlete relationship influences athlete burnout through team efficacy. This finding demonstrates that the coach–athlete relationship and team efficacy play important roles in preventing athlete burnout. The importance of the coach–athlete relationship and team efficacy has been confirmed by a number of studies (Mageau and Vallerand, 2003; Jowett and Poczwardowski, 2007; Høigaard et al., 2015; Huh and Choi, 2017; Jang and Seong, 2017; Choi and Cho, 2019; Stephen et al., 2022). Effective communication is one of the most important considerations when attempting to accomplish team members’ goals. When communication is bidirectional, accurate information can be conveyed to another person. From this viewpoint, Huh and Choi (2017) stressed that constant interaction between coaches and athletes is required to achieve goals desired by individuals or teams in sports. Therefore, when a high-quality relationship between coaches and athletes is maintained through effective communication, it is possible to increase team efficacy and strengthen athletes’ abilities to implement tactics through cohesive teamwork.

A recent study by Choi and Jung (2020) emphasized the importance of improving the coach–athlete relationship and maintaining a good coach–athlete relationship to prevent athlete burnout. In actual competition and training situations, a poor coach–athlete relationship (i.e., lack of closeness, commitment, and complementarity) has been shown to be positively associated with athlete burnout, whereas a good one is known to decrease the level of athlete burnout (Isoard-Gautheur et al., 2016; Davis et al., 2018). The quality of the coach–athlete relationship can be improved by effectively using emotion regulation strategies related to interpersonal relationships. Therefore, from an interactionist perspective, to form desired emotions or provide each other with desired emotional states, coaches and athletes need to use emotion regulation strategies effectively. Coaches can decrease athlete burnout by providing a positive motivational environment.

In this study, team efficacy negatively affected athlete burnout, suggesting that it reflects a positive perception of good feelings or attitudes among team members.

Haikarainen and Stenberg (2018) expected that a good coach–athlete relationship would act as a buffer for the negative aspects of teammate burnout. They hypothesized that a higher level of perceived teammate burnout is associated with a higher level of athlete burnout symptoms and that a better coach–athlete relationship is more likely to play a mediating role in the relationship between perceived teammate burnout and athlete burnout. However, their study failed to show a mediating effect of the coach–athlete relationship. Nevertheless, the study findings described above suggest that the quality of the coach–athlete relationship may depend on the stress level of individual athletes, and may influence athletes constituting a sports team, thereby playing an important role in reducing or preventing athlete burnout. Therefore, athletes should recognize the importance of team efficacy rather than rely entirely on their individual confidence and should try to increase their belief in their team’s capabilities by frequently experiencing accomplishments in sports games.

Some researchers (Day et al., 2009; Isoard-Gautheur et al., 2016; Park and Huh, 2018) emphasized the importance of team efficacy as a predictor of burnout prevention, claiming that research should be conducted from various perspectives to enhance team efficacy. A previous study by Davis et al. (2018) presented relevant empirical results. The study reported that athlete burnout decreased when teammates exchanged emotions and shared perceptions about the training. As shown by the findings of previous studies, it is important to understand the factors that proactively prevent athlete burnout because understanding these factors can improve athletes’ athletic performance, emotional stability, health, and engagement, and maximize outcomes. Above all, there is a need to provide educational programs to help coaches and athletes understand the burnout prevention process, recognize the importance of the effects of burnout prevention, and gain access to methods for burnout prevention. This is expected to subsequently contribute to strengthen team members’ motivation and create advanced strategies to improve performance (outcomes).

Finally, with respect to autonomy support, perceived autonomy support had a significant negative effect on aggressive behavior through team efficacy. In this study, the estimate for the indirect (mediated) effect of autonomy support on aggressive behavior was −0.171. This indicates that as autonomy support increases, aggressive behavior decreases by 0.171 times due to the indirect (mediated) effect of autonomy support on aggressive behavior. As mentioned earlier, autonomy-supportive behavior plays an important role in improving team efficacy. As autonomy-supportive coaching behavior is intended to achieve goals desired by individual athletes or the team, it influences athletes’ athletic development. From a future-oriented perspective, it is expected to contribute to the formation of a more positive coach–athlete relationship and a strong emotional bond between coaches and athletes. Álvarez et al. (2009) argued that autonomy-supportive coaching behavior is a positive predictor of athletes’ emotions. Thus, in competitive sports settings, such behavior can be viewed as an important antecedent variable that can effectively increase the level of team efficacy and may therefore be used as an effective strategy to improve the performance of individual players or teams.

Athletes in teams with high team efficacy are expected to reduce or suppress individual aggressive behavior for team success by controlling themselves or putting their team before themselves, even in emotionally exciting situations. As it is strongly expected that athletes’ aggressive behavior during games may be caused by the latent perception that aggression is a way to achieve the goal of winning a game, the perception of high team efficacy is expected to suppress aggressive behavior.

Athletes’ aggressive behavior is closely related to sports performance. In particular, athletes’ low expectations about their individual ability and performance may cause their aggressive and violent behavior, and this type of behavior may be regarded as aggressive behavior due to cognitive biases. Brubacher et al. (2018) reported that a higher level of self-efficacy is associated with a lower level of aggressiveness, and a lower sense of belonging is linked to a higher level of aggressive behavior. Therefore, if team sports athletes have positive thoughts based on their high self-efficacy, respect other teams’ athletes, and have a fair play attitude (e.g., humility about victory, generosity during games, and graciousness in defeat), they will prioritize behaviors for the team in such a way that is reflected by the frequently quoted phrase: “No one player is bigger than the team.” This will lead to a reduction in aggressive behavior.

The results of this study revealed that perceived coach autonomy support plays an important role in enhancing team efficacy, and that a high level of team efficacy can reduce athletes’ aggressive behavior. These findings suggest the need to pay more attention to team efficacy as a means of reducing conflicts among members.

## Conclusion and suggestions

The basic principle of team sports is to prioritize the team over individual athletes and win or lose games as a team. In this process, members collaborate to achieve personal growth through competition. Teamwork is essential to team sports. Teams with good teamwork processes produce surprisingly good results. Team members should cooperate mentally and technically toward common goals; in terms of this interaction, it is very important for them to have the intention of understanding other members and a sincere attitude of listening to and respecting them.

Therefore, autonomy-supportive coaching behaviors can improve the quality of relationships among members and strengthen team efficacy, allowing team members to establish effective team strategies and expect successful outcomes such as victory. From this perspective, this study investigated the mediating effects of the coach–athlete relationship and team efficacy on the relationship between perceived coach autonomy support and burnout in team sports athletes. The results showed that perceived coach autonomy support significantly affected athlete burnout through the coach–athlete relationship and team efficacy. Based on these results, the actual causes of athlete burnout and factors that can reduce or prevent athlete burnout were identified. Also, it was also found that in team sports, the role of team efficacy is more important than coach–athlete relationship in decreasing athletes’ aggressive behavior. Nevertheless, I believe that understanding and interpreting athlete burnout solely from psychological and social approaches is insufficient. Therefore, it is deemed very important to study athlete burnout from a new perspective, namely the neuroscientific aspect, to elucidate the neuroscientific mechanisms or related factors (Davidson, 2002; Bermond, 2008).

Additionally, in team sports settings, individual athlete burnout may manifest as team burnout. Therefore, in follow-up studies, it is necessary to measure burnout by distinguishing between athlete burnout and team burnout. Furthermore, an analysis of the relationship between burnout and other factors such as the coach–athlete relationship, conflicts among members, team efficacy, and team cohesion is also necessary to present a broad range of potential directions of burnout. Lastly, based on the results of this study, more in-depth research on team sports is required.

## Data availability statement

The datasets presented in this study can be found in online repositories. The names of the repository/repositories and accession number(s) can be found in the article/supplementary material.

## Ethics statement

The studies involving humans were approved by institutional board of Kangwon National University. The studies were conducted in accordance with the local legislation and institutional requirements. The participants provided their written informed consent to participate in this study.

## Author contributions

S-KK: Writing - original draft, Writing – review & editing, Data curation, Methodology, Conceptualization, Funding acquisition. HC: Writing – original draft, Writing – review & editing, Data curation, Methodology, Formal analysis.
